# Fungal Metabolites with Antagonistic Activity against Fungi of Lithic Substrata

**DOI:** 10.3390/biom11020295

**Published:** 2021-02-16

**Authors:** Marco Masi, Mariagioia Petraretti, Antonino De Natale, Antonino Pollio, Antonio Evidente

**Affiliations:** 1 Dipartimento di Scienze Chimiche, Università di Napoli Federico II, Complesso Universitario Monte S. Angelo, Via Cintia 4, 80126 Napoli, Italy; evidente@unina.it; 2 Dipartimento di Biologia, Università di Napoli Federico II, Complesso Universitario Monte S. Angelo, Via Cintia 4, 80126 Napoli, Italy; mariagioia.petraretti@unina.it (M.P.); denatale@unina.it (A.D.N.); antonino.pollio@unina.it (A.P.)

**Keywords:** fungal metabolites, antifungal activity, cultural heritage, fungi, stone biodeterioration, stone biodegradation

## Abstract

Fungi are among the biotic agents that can cause deterioration of building stones and cultural heritage. The most common methods used to control fungal spread and growth are based on chemical pesticides. However, the massive use of these synthetic chemicals produces heavy environmental pollution and risk to human and animal health. Furthermore, their use is time dependent and relies on the repetition of treatments, which increases the possibility of altering building stones and culture heritage through environmental contamination. One alternative is the use of natural products with high antifungal activity, which can result in reduced toxicity and deterioration of archeological remains. Recently, three fungal strains, namely *Aspergillus niger, Alternaria alternata* and *Fusarium oxysporum,* were isolated as damaging agents from the external tuff wall of the Roman remains “Villa of Poppea” in Oplontis, Naples, Italy. In this manuscript, three selected fungal metabolites, namely cyclopaldic acid, cavoxin and *epi*-epoformin, produced by fungi pathogenic for forest plants, were evaluated as potential antifungal compounds against the above fungi. Cavoxin and *epi*-epoformin showed antifungal activity against *Asperigillus niger* and *Fusarium oxysporum*, while cyclopaldic acid showed no activity when tested on the three fungi. The same antifungal activity was observed in vitro experiments on infected stones of the Neapolitan yellow tuff (NYT), a volcanic lithotype widely diffused in the archeological sites of Campania, Italy. This study represents a first step in the use of these two fungal metabolites to allow better preservation of artworks and to guarantee the conditions suitable for their conservation.

## 1. Introduction

Fungi are among the major agents of microbial deterioration of building stones and cultural heritage. Deterioration caused by fungal colonization involves aesthetic, physical and chemical damage of stone surfaces, which, in most cases, take place simultaneously [1]. Besides aesthetic damage due to color change, black spots and patina formation, fungi can deeply colonize cracks and fissurations because of the extraordinary penetrating power of fungal hyphae into the substratum, causing breaking and lesions in artwork. Fungi are able to excrete a large variety of organic acids that are able to act as metal-chelators, mediating the precipitation of secondary minerals like carbonates, oxalates and phosphates, which can cause blistering, scaling and granular disintegration, leading to stone decay [2]. For these reasons, it is important to find solutions to the deterioration of stone surfaces that do not compromise the readability and structure of artworks.

Synthetic pesticides, including fungicides, are massively used in agriculture, forests, parks, and archeological areas. The heavy pollution, essentially of soil and water, due to the worldwide use of these chemicals has prompted the search for eco-friendly alternatives based on bioactive natural substance formulations. This also satisfies the requests of people and authorities who ask for safe products to avoid the contamination of food and lower the risk to human and animal health [3,4,5]. 

Plants, microorganisms, lichens, and algae are producers of metabolites possessing diverse biological activities, such as phytotoxic, antiviral, anticancer, antitumor, algicide, enzyme-inhibiting, immunostimulant, antiplatelet aggregation, cytotoxic, antiplasmodium, antibacterial, and antifungal activities [6,7,8,9]. Several secondary metabolites with antimicrobial activity have already been isolated from different fungi [10,11,12,13,14]. These metabolites belong to diverse structural classes of naturally occurring compounds (e.g., alkaloids, anthraquinones, poliketides, terpenes, steroids), and most of them possess original modes of action to overcome antimicrobial resistance [15,16,17,18,19]. 

Recently, three fungal strains, namely *Aspergillus niger, Alternaria alternata* and *Fusarium oxysporum,* were isolated as damaging agents from the external tuff wall of the Roman remains, “Villa of Poppea” in Oplontis, Naples, Italy. 

This manuscript reports the assessment of in vitro systems to study the early steps of fungal colonization of stone (Neapolitan yellow tuff, a volcanic lithotype widely diffused in the archeological sites of Campania, Italy) and to test the antifungal properties of three fungal metabolites, namely cavoxin, *epi*-epoformin and cyclopaldic acid, for their potential fungicidal activity.

## 2. Materials and Methods

### 2.1. General Chemical Procedure 

Electrospray ionization mass spectrometry (ESI MS) and liquid chromatography/mass spectrometry (LC/MS) analyses were performed using an LC/MS TOF system Agilent 6230B (Agilent Technologies, Milan, Italy), HPLC 1260 Infinity. A Phenomenex (Bologna, Italy) Luna (C18 (2) 5 mm, 150–4.6 mm column) was used to perform the high-performance liquid chromatography (HPLC) separations. Bruker 400 Anova Advance (Karlsruhe, Germany) was used to record the ^1^H NMR spectra at 400 MHz in CDCl_3_ at 298 K. Analytical, preparative, and reverse-phase thin-layer chromatography (TLC) were carried out on silica gel (Merck, Kieselgel 60, F_254_, 0.25, 0.5 mm, and RP-18 F_254s_, respectively) plates (Merck, Darmstadt, Germany). The spots were visualized as previously described [20]. Column chromatography (CC) was performed using silica gel (Kieselgel 60, 0.063–0.200 mm) and C_18_-reversed phase silica gel (230–400 mesh) (Merck, Darmstadt, Germany). Sigma-Aldrich Co. (St. Louis, MO, USA) supplied all the reagents and the solvents. 

### 2.2. Fungal Metabolites

Cavoxin was obtained as previously reported [21] from *Phoma cava* (CBS (Centraalbureau voor Schimmelcultures), The Netherlands, 535.66). The fungus was grown in flasks containing 300 mL of a semisynthetic liquid medium incubated at 25 °C and 200 rpm for 5 days. The cultures were filtered, and the filtrate was lyophilized. The solid residue corresponding to 9 L of culture filtrate was dissolved in distilled water and extracted with CHCl_3_. The organic extract was chromatographed on a Sephadex LH-20 column eluted with CHCl_3_-*i*PrOH (9:1, *v*/*v*), obtaining cavoxin as a homogeneous oil, which was crystallized from EtOAc-petroleum ether (1:1, *v*/*v*) as pale-yellow needles (979 mg). 

Cyclopaldic acid was obtained from *Seirdium cupressi* as previously reported [22]. The strain of *S. cupressi* was isolated in Kos (Greece) and deposited in the collection of Dipartimento di Patologia Vegetale, University of Bari, Italy, as DPG10. It was grown as stationary culture in 1 L Roux flasks containing 150 mL Czapek’s medium with the addition of 2% corn meal and incubated at 23 °C for 30 days in the dark. The culture filtrates were acidified to pH 4 and extracted with *t*-butylmethyl ether. The combined organic extracts afforded an oil, which was washed with CHCl_3_, leaving a white amorphous substance. The latter gave cyclopaldic acid (750 mg) by crystallization from MeOH-CHCl_3_ (1:1, *v/v*). 

*epi*-Epoformin was obtained from *Diplodia quercivora* as previously described [23]. A strain of *D. quercivora* was isolated from a symptomatic branch of *Quercus canariensis* in a natural area in Tunisia and deposited in the collection of the Dipartimento di Agraria, University of Sassari, Italy, as BL9. The fungus was grown in 2 L Erlenmeyer flasks containing 400 mL of Czapek medium amended with corn meal and incubated at 25 °C for 3 weeks in darkness. The culture filtrates (6.7 L) were acidified to pH 4 and extracted exhaustively with EtOAc. The organic extracts were purified on silica gel and successively on reverse-phase column chromatography, yielding *epi*-epoformin (276.1 mg) as a white solid. 

The purity of cavoxin, cyclopaldic acid, and *epi*-epoformin (>98%) was ascertained by HPLC, ^1^H NMR and ESI MS spectra. 

### 2.3. Antifungal Test-Well Diffusion Assay

A conidia suspension of the test fungi was obtained from a 6-day-old colony grown in solid-medium potato dextrose agar (PDA) treated with PBS-Tween20 solution 0.5%, by scraping from the agar surface with a sterile spatula. Conidia were suspended in physiological solution (0.9% NaCl) to a final concentration of 1 × 10^6^ conidia/mL. 50 µL of the final conidia suspension was spread onto the PDA until the suspension was completely absorbed. Then, filter paper discs 13 mm in diameter, previously absorbed with 20 µL of each chemical compound at the desired concentration, were placed on the agar surface. Finally, the Petri dishes were incubated at 22 ± 2 °C in darkness for 3 days with control fixed every 24 h. The natural biocides selected for this study were cavoxin, cyclopaldic acid and *epi*-epoformin. All of these were tested in several concentrations, namely 0.25, 0.50 and 1.0 mg/L. Then, the eventual diameters of inhibition growth zones were measured. Pentachloronitrobenzene (PCNB) was used as positive control [24], while empty filter paper discs were used as negative control. Filter paper discs absorbed with 20 µL of methanol were used to verify the eventual interferation with fungal growth.

### 2.4. Lithotypes and Microorganisms

The selected fungal strains were *Aspergillus niger* (008f), *Alternaria alternata* (015f) and *Fusarium oxysporum* (014f)*,* obtained from ACUF, Algal Collection of University of Naples Federico II, Italy. A new section of this collection, which has been traditionally devoted to the maintenance of aero-terrestrial microalgal and cyanobacteria strains [25], was recently created to keep fungal strains that have been isolated directly from archeological sites in Campania. In particular, these strains were collected from the external tuff walls of “Villa of Poppea” in Oplontis (Naples, Italy). The identification of fungal strains was assessed on the basis of morphological observations coupled with molecular analysis. DNA was extracted with a modified Doyle and Doyle DNA extraction protocol [26] and used for a polymerase chain reaction using ITS spacers as target primer (primer forward 5′-TCCGTAGGTGAACCTGCGG-3′; primer reverse 5’-TTCAAAGATTCGATGATTCAC-3’). The PCR products were evaluated on agarose gel in an electrophoretic run and purified using a QIAquick^®^ PCR Purification kit (Qiagen Inc., Valencia, CA, USA). The sequence reaction was obtained with BigDye Terminator Cycle Sequencing technology (Applied Biosystems, Foster City, CA, USA), using the amplification primers as the sequencing primers, and purified using the Agencourt CleanSEQ Dye terminator removal kit (Agencourt Bioscience Corporation, 500 Cummins Center, Suite 2450, Beverly, MA, USA) and a robotic station Biomek FX (Beckman Coulter, Fullerton, CA, USA). The product was analyzed by an Automated Capillary Electrophoresis Sequencer 3730 DNA Analyzer (Applied Biosystems). Nucleotide sequence similarity was determined using BLAST version 2.0 (https://blast.ncbi.nlm.nih.gov (accessed on 26 January 2021)). The obtained sequences were identified as *Aspergillus niger, Alternaria alternata* and *Fusarium oxysporum.* The stone selected for the in vitro biodeterioration test was the Neapolitan yellow tuff (NYT), a volcanic lithotype widely diffused in the archeological sites of Campania, which is known for its porosity and great water absorption coefficient, both of which support the colonization of microorganisms [27]. 

### 2.5. In Vitro Biodeterioration Test

In order to reproduce the biological damage caused by fungi on stone, a series of in vitro tests was performed by inoculating the selected fungal strains on stone. NYT tiles (3 × 3 cm) were placed in Petri glass dishes and inoculated with a standardized inoculum of the test fungi [28]. The dishes were incubated at 22 ± 2 °C for 20 days with 90–100% of relative humidity to reproduce the environment in which the fungal strains occurred. For each fungal strain, four glass Petri dishes were prepared containing three tuff tiles. In order to evaluate the potential degradation activity of metabolites considered in this study, NYT tiles were also inoculated with fungi together with the tested metabolites at a concentration of 1 mg/mL. To evaluate the fungal growth, the experiments were monitored for 20 days. Once the measurements were taken, the tuff tiles were discarded. Each set of measurements was performed for 20 days at an interval of 5 days in the following way: (1) quantification of the fungal covered area on tiles by recording digital images; (2) measurement of the fungal thickness on tiles with a metallurgical microscope with an objective 6.5×.

### 2.6. Laboratory Strains and Culturing Conditions 

All the NYT tiles used in the experiments were washed, dried, and displaced in triplicate in Petri glass dishes. On the bottom of each plate was placed a filter paper disc flooded with sterile distilled water in order to guarantee 90–100% of relative humidity. The tiles were then inoculated with 70 µL of sterile Bold’s Basal Medium (BBM) [29] with 12 g/L of sucrose added [30] in order to provide an equal starting nutrient source for all the fungal strains. For the experiment aimed at evaluating the antifungal activity of metabolites considered in this study, together with fungal growth medium, an amount of 70 µL of the metabolites at a concentration of 1 mg/mL was inoculated. In all the experiments, for each selected strain, an inoculum of 5000 conidia suspended in 5 µL of sterile distilled water was inoculated in the middle of each tile, as previously reported [28].

Two other glass chambers were prepared with tuff tiles watered with (1) distilled water instead of the nutritive medium and (2) methanol and kept until the end of the experiment as controls. To evaluate the fungal growth, every 5 days three tuff tiles per strain from each of the four glass chambers were analyzed, and once the measurements were taken, they were discarded. During the whole time of observation, the potential occurrence of bacteria was microscopically checked by sampling at regular times the fungal population growing on the tiles. No significant bacterial growth was observed throughout the experiments. All the described procedures took place under a laminar flow hood, and all the materials used were tindalized.

### 2.7. Evaluation of Fungal Growth

The fungal growth and thickness on the tuff tiles were determined after 5, 10, 15 and 20 days of incubation. Tuff tiles were photographed and recorded every 5 days with a digital camera (Nikon D5100 50 mm objective). The lens of the camera was always kept at the same distance from the samples inside a laminar flow hood, with the lids of the Petri dishes off. The digitized images were analyzed using Trainable Weka Segmentation [31,32], a plugin of open source software of digital image analysis, namely Fiji [33]. Colony growth on the tuff tiles was also determined by measuring fungal thickness on the same samples used for the quantification of covered areas at each incubation period. To determine the thickness values, the procedure was followed according to Bakke and Olsson [34], with a metallurgical microscope (Leitz Wetzlar Ortholux Microscope) with an objective 6.5×. In this set of experiments, each tile was virtually divided into three zones, ranging from the middle to the external borders of the tile, as described by Del Mondo et al. [28]; these zones were central, median and distal. The results obtained by the triplicates for any given set of measurements for both the thickness and surface data were used as means for each observation point and then plotted with their respective standard errors.

### 2.8. Statistical Analysis

Statistical analyses were carried out by two-way analysis of variance (ANOVA), and means were compared by Dunnett’s test, using Prism software, from three independent replicate values. The value of *p* ≤ 0.05 was considered statistically significant, as noted by an asterisk accompanying means in figures.

## 3. Results and Discussion

**Figure 1:**
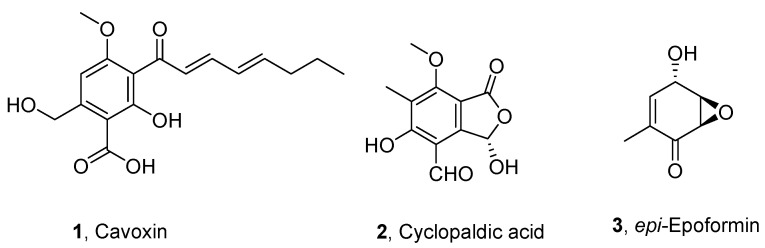
Structures of cavoxin, cyclopaldic acid and *epi*-epoformin (**1**–**3**).

The fungal metabolites were selected to assay their antifungal activity among a plethora of natural substances isolated and purified in our laboratory at the Department of Chemical Science of University of Naples Federico II in Naples, Italy. In the literature data, the most promising appeared to be the three fungal metabolites cavoxin [21], cyclopaldic acid [22], and *epi*-epoformin [23] (**1**–**3**, respectively, in Figure 1).

The three metabolites were isolated and purified as phytotoxins from pathogens of forest plants as *Phoma cava*, *Seiridium cupressi* and *Diplodia quercivora*, which are the causal agent of chestnut (*Castanea sativa*) and cypress (*Cupressus semperirens* L.) canker diseases [21,22,23]. In particular, cavoxin showed antifungal activity against *Aspergillus niger* and *Penicillium roqueforti*, and thus was bioformulated in PBS for intelligent food packaging to protect bread bakery products [35]. Cyclopaldic acid was active against *Botrytis cinerea*, *Fusarium solani* and *Geotrichum candidum* in a structure activity relationships study [36]. Furthermore, cyclopaldic acid and *epi*-epoformin showed antifungal activity against *Puccinia triticina* and *Uromyces pisi*, two rusts pathogens for *Pisum sativum* and other legumes [37].

**Figure 2:**
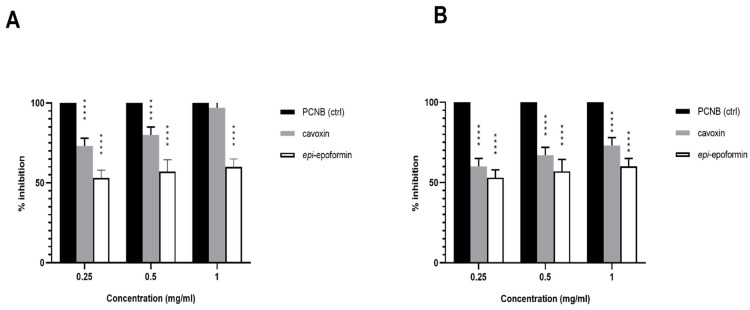
Percentage of inhibition, normalized to the positive control (PCNB) (black bar), of cavoxin (grey bar) and *epi*-epoformin (white bar), against *A. niger* (**A**) and *F. oxysporum* (**B**). Data shown are means ± SD of three independent experiments. **** indicates *p* < 0.0001.

**Figure 3:**
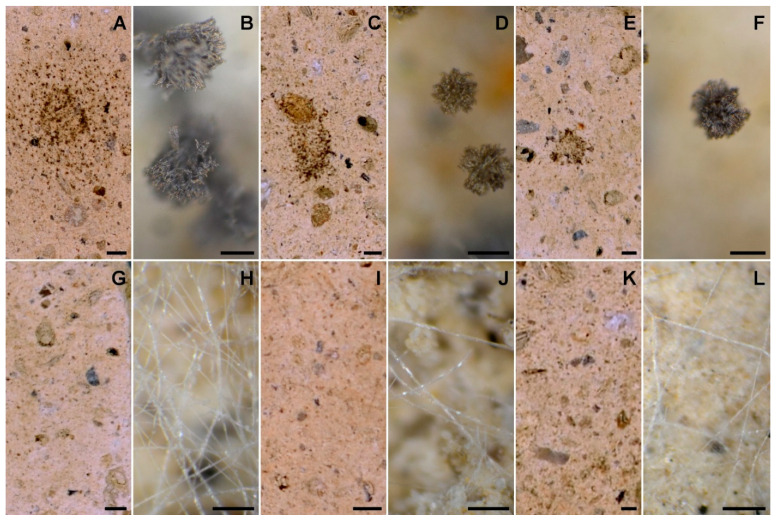
Development of fungal growth on tuff tiles at 20 days after inoculation of *A. niger* (**A**: control, **C**: with cavoxin, and **E**: with *epi*-epoformin) and *F. oxysporum* (**G**: control, **I**: with cavoxin, and **K**: with *epi*-epoformin) coupled with the respective metallurgical microscopy images (**B**, **D**, and **F** for *A*. *niger* and **H**, **J**, and **L** for *F. oxysporum*); scale bar on tuff tiles is 2 mm, scale bar for metallurgical microscopy is 50 µm.

The antifungal activity of cavoxin, cyclopaldic acid and *epi*-epoformin was first tested against *A. niger, A. alternata* and *F. oxysporum* by the paper disk diffusion assay. Cavoxin and *epi*-epoformin inhibited *A. niger* and *F. oxysporum*, while cyclopaldic acid did not show any activity on the three strains. In particular, cavoxin and *epi*-epoformin inhibited the growth of *A. niger* and *F. oxysporum* after 72 h at different concentrations, while no activity was shown against *A. alternata*. For this reason, the strain was discarded from in vitro analysis. The sensitivity of *A. niger* and *F. oxysporum* was concentration-dependent, and cavoxin was shown to be more effective than *epi*-epoformin (Figure 2). Digital image analysis complemented with metallurgical microscopy was employed for evaluating the growth of fungal strains on tuff tiles and the potential biocide activity of cavoxin and *epi*-epoformin. The condition selected for the experiments supported a visible fungal growth within the time course of the experiments (20 days). The development of *A. niger* and *F. oxysporum* growth on tuff tiles, coupled with the respective metallurgical microscopy images, after 20 days both in presence and absence of the tested compounds (cavoxin and *epi*-epoformin) are shown in Figure 3.

**Figure 4:**
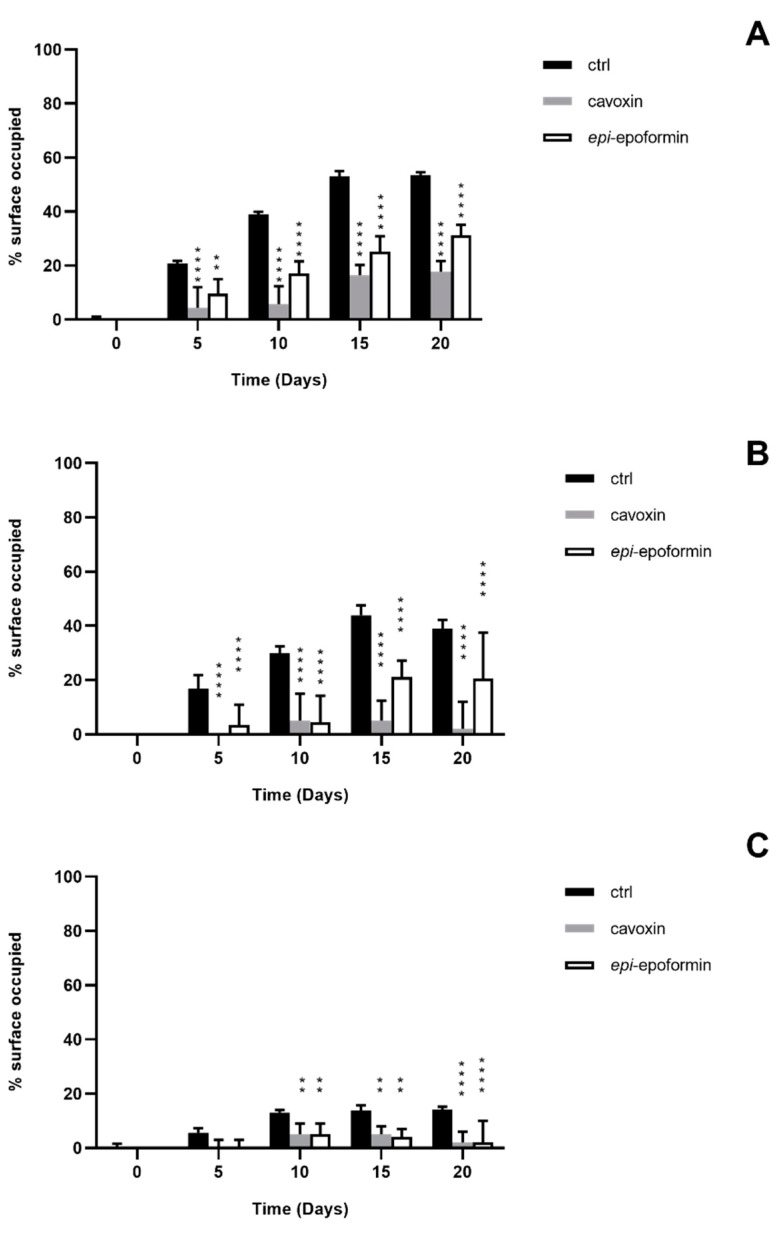
Percentage of surface occupied by *F. oxysporum* on central region (**A**), median region (**B**), and distal region (**C**) of the tuff tile. Untrated (black bar), trated with cavoxin (grey bar), treated with *epi*-epoformin (white bar). Data shown are means ± SD of three independent experiments. ** indicates *p* < 0.005, and **** indicates *p* < 0.0001.

**Figure 5:**
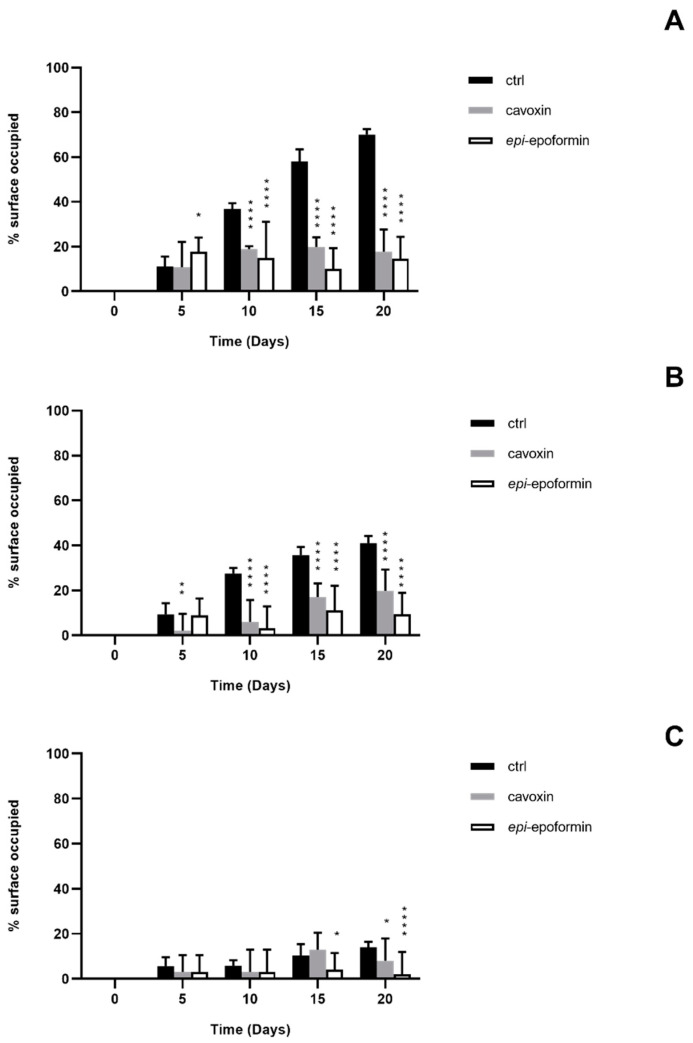
Percentage of surface occupied by *A. niger* on central region (**A**), median region (**B**), and distal region (**C**) of the tuff tile. Untreated (black bar), treated with cavoxin (grey bar), treated with *epi*-epoformin (white bar). Data shown are means ± SD of three independent experiments. * indicates *p* < 0.05, ** indicates *p* < 0.005, and **** indicates *p* < 0.0001.

**Figure 6:**
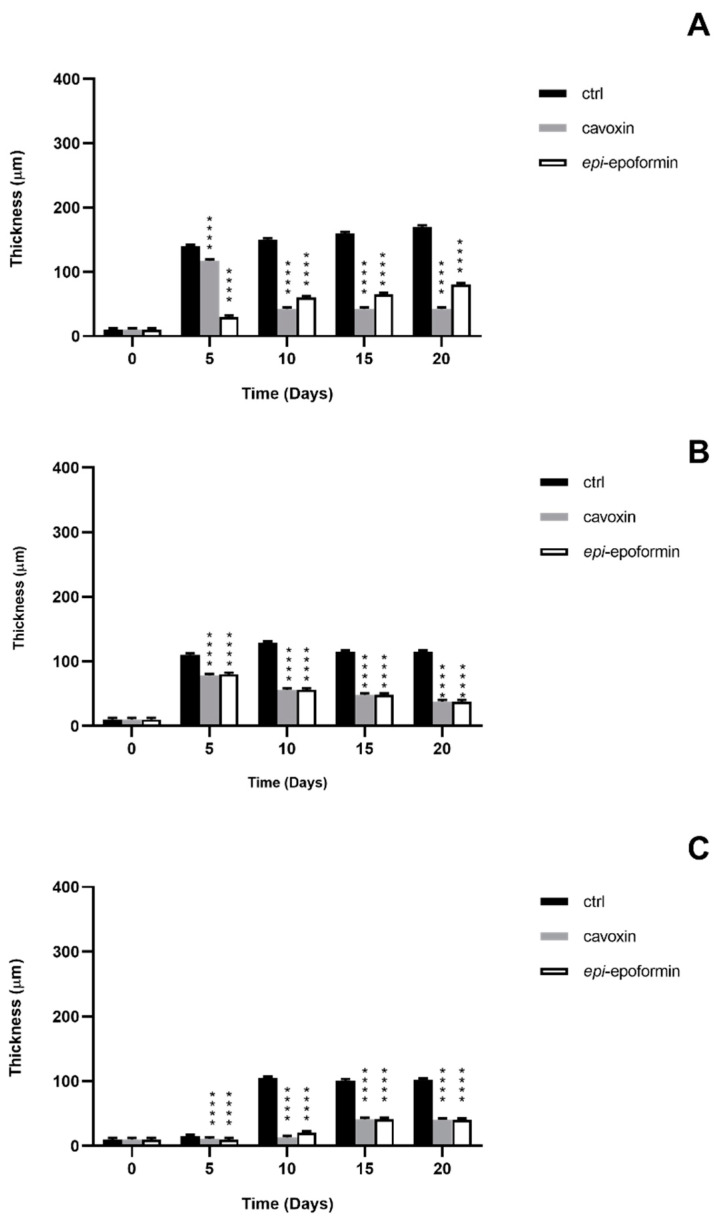
Thickness of *F. oxysporum* on central region (**A**), median region (**B**), and distal region (**C**) of the tuff tile. Untreated (black bar), treated with cavoxin (grey bar), treated with *epi*-epoformin (white bar). Data shown are means ± SD of three independent experiments. **** indicates *p* < 0.0001.

**Figure 7:**
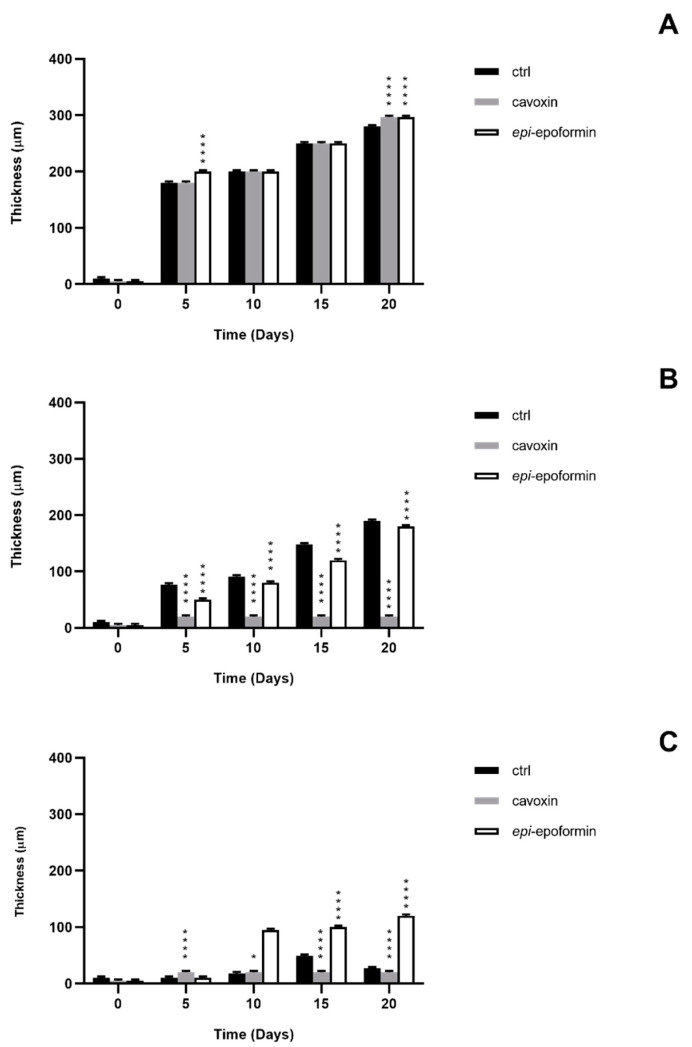
Thickness of *A*. *niger* on central region (**A**), median region (**B**), and distal region (**C**) of the tuff tile. Untreated (black bar), treated with cavoxin (grey bar), treated with *epi*-epoformin (white bar). Data shown are means ± SD of three independent experiments. * indicates *p* < 0.05, **** indicates *p* < 0.0001.

In controls, *F. oxysporum* and *A. niger* formed compact mycelia that had partly overgrown the surface of tiles. A radial development of the fungal mycelia was observed, and it was possible to establish a temporal sequence of tile colonization, with fungal hyphae progressively extending from the zone of the inoculum to the median and distal regions of the tiles. At later stages of colonization, *F. oxysporum* and *A. niger* respectively occupied about 60% and 70% of the tile surfaces (Figure 4 and Figure 5). A corresponding increase of thickness was also observed, but *F. oxisporum* attained an average thickness of about 150 µm in central and median regions of the tiles, whereas *A. niger* achieved about 300 µm in the central region and 200 µm in the medial one (Figure 6 and Figure 7).

The ability of *Aspergillus* sp. to rapidly produce large biomasses firmly adhering to substratum has been reported, as has the ability to withstand different humidity and temperature conditions [38,39]. Both fungi showed a very reduced thickness in distal regions of the tiles, where the hyphal network presented numerous large voids (not shown), as previously reported [28]. The treatment with cavoxin and *epi*-epoformin was very effective against the growth of *A. niger* and *F. oxysporum* on tuff tiles, drastically reducing the colonization of both fungi. In the treated tiles, both surface coverage and thickness decreased in all the regions (central, medal, distal) of the tiles. In the experiments with *F. oxysporum*, the maximum superficial coverage observed corresponded to about 30% for tiles treated with *epi*-epoformin and was reduced to 20% for those with cavoxin. Similar results were obtained with *A. niger*, whose coverage did not exceed a 20% of tile surface in the presence of each compound.

Laboratory-based stone colonization experiments using different fungal isolates as target organisms are well-established tests to assess the efficacy of the control techniques implemented for the conservation of stone artworks [40,41]. Different approaches have been attempted to eradicate the biofilms growing on artworks, ranging from ultraviolet rays and laser cleaning to ice cleaning systems and microwaves [42]. In addition, the application of protective products and/or hydrophobic agents does not prevent stone colonization by fungi, and the combined application of biocides is required [43]. In terms of the use of biocides, by far the most frequently adopted strategy of control, synthetic chemicals or mixtures derived from plant preparations (essential oils or water extracts) have been frequently tested [44]. Both can be effective but present some points of weakness. They have a time-limited action on biofilms, depending on environmental parameters such as humidity and temperature [45]. Moreover, it is well known that the application of many biocides on stone surfaces may increase stone tertiary bioreceptivity [46] due to their composition, which might be utilized by microorganisms as nitrogen and carbon sources [47]. This is one of the reasons why the use of traditional biocides to reduce the phenomenon of biodeterioration of stone monuments is increasingly discouraged [44]. In this respect, the investigation of fungal active metabolites opens a promising avenue of research. Due to the amazing number of chemically different substances produced by fungi, it is possible to design biocides targeting specific microbial communities, using combinations of antifungal natural products that selectively inhibit the growth of individual fungal species within the biofilm.

## 4. Conclusions

This study represents a first step in the use of fungal metabolites to allow a better preservation of artwork and to guarantee the conditions suitable for their conservation. The results obtained with *epi*-epoformin and cavoxin confirm that fungi are an untapped source of effective substances to halt or reduce biodeterioration. However, a prerequisite of their application will be to assess their possible detrimental effects on different lithic materials as well as environmental compatibility, in order to develop an eco-friendly system of biofilm control that is respectful of the uniqueness of each artwork.
